# Going to the COVID-19 Gemba: Using observation and high reliability strategies to achieve safety in a time of crisis

**DOI:** 10.1017/cem.2020.380

**Published:** 2020-04-24

**Authors:** Jennifer Thull-Freedman, Shawn Mondoux, Antonia Stang, Lucas B. Chartier

**Affiliations:** * University of Calgary Cumming School of Medicine, Departments of Pediatrics and Emergency Medicine, Calgary, AB; † Alberta Children's Hospital Research Institute, Calgary, AB; ‡ McMaster University, Department of Medicine, Division of Emergency Medicine, Hamilton, ON; § St. Joseph's Healthcare Emergency Department, Hamilton, ON; ¶ Department of Medicine, Division of Emergency Medicine, University of Toronto, Toronto, ON; ** University Health Network Emergency Department, Toronto, ON

**Keywords:** COVID-19, emergency department, high reliability organizing, pandemic, patient safety

Implementation of high reliability principles in healthcare delivery is recognized as an effective strategy for reducing harm to patients and healthcare workers.^1,2,3^ With the coronavirus disease 2019 (COVID-19) pandemic upon us, our emergency departments (EDs) are facing an unprecedented safety threat. How does a high reliability ED function during a pandemic, and what are the most important strategies for keeping ourselves and our patients safe?

Historically, in medicine, safety was viewed as mostly an individual responsibility, and errors were viewed as human failings. This approach, described by James Reason as the “person approach,” has many shortcomings.^4^ Importantly, it does not identify and address system issues that predispose to error. Conversely, the “system approach” focuses on understanding the conditions in which individuals work, identifying root causes of errors, and developing defenses. High reliability organizations (HROs) recognize that humans by nature are fallible, and therefore they create systems to recognize failure at early stages and contain damage.^4^ As individual healthcare providers facing the threat of a deadly pandemic, we seem to inherently recognize that we must rely not only on our individual dedication, but also, more importantly, on the reliability of our teams and our systems to prevent unnecessary harm from reaching ourselves and our patients.

An HRO is one that is able to achieve very low rates of error, despite operating in complex and high-risk circumstances.^1^ In their landmark book, *Managing the Unexpected*, Weick and Sutcliffe outline five principles of anticipation and containment that define organizations that are able to achieve low rates of error, despite highly complex, high-stake environments. They assert that to avoid error, organizations must create a mindful infrastructure that tracks small failures, resists oversimplification, remains sensitive to operations, maintains capabilities for resilience, and takes advantage of shifting locations of expertise.^1^ Recently, the term “high reliability *organizing*” has been used in addition to “high reliability *organizations*” to emphasize the dynamic nature of reliably averting failure. Weick asserts that reliability is both a moving target and a dynamic non-event.^5^ A dynamic non-event is never permanently achieved; rather, ongoing vigilance is required to prevent errors from occurring.^6^ The challenge we will face in the coming months will be about how to prepare our EDs to reliably produce non-events in the setting of a pandemic. To do this, we can direct our energies to the five principles of HROs outlined by Weick and Sutcliff:

1. **Preoccupation with failure**
  Teams that are preoccupied with failure recognize that even the best-made plans are at risk for failing, and they vigilantly assess for potential lapses in reliability. With this mindset, everyone has a responsibility for both doing their work and identifying threats to reliable system functioning. Early warning signals are spotted, and psychological safety allows team members to report and share errors. While recognizing the potential for failure, teams strive to improve and do not accept dismissive attitudes such as “we'll never be perfect.”
2. **Reluctance to simplify interpretation**
  Teams do not make hasty assessments of problems. Diligent investigation allows continuing learning. Teams use a systematic approach, such as root-cause analysis, to understand errors and deviations.^7^ Cognitive biases are recognized, such as the availability bias of over-attributing significance to similarities with past experiences.
3. **Sensitivity to operations**
  Teams understand potential failure modes in operations and anticipate how changes in one part of a system may affect another. Critical processes are observed and measured. Leaders are present where work is occurring and understand the actual work being performed rather than just the intended work.
4. **Commitment to Resilience**
  Teams are engaged in safety and ready to respond quickly when there is deviation from optimal performance. They are able to continue effective operations in the presence of stress. Teams continuously learn from errors and adapt.
5. **Deference to expertise**
  Decisions are made by those with the most relevant experience in the area. Rather than assuming that hospital administrators bear responsibility for system issues, frontline staff play a key role in contributing to improvements. Leaders acknowledge the expertise of frontline staff. Interprofessional teams share knowledge without being limited by hierarchical relationships. Psychological safety allows open communication and productive conflict.

In the context of pandemic preparation, implementation of high reliability methods in the ED will include multiple strategies to anticipate and mitigate threats. These may include simulation and observation of practices; development of checklists; diligent reporting of errors, near-misses, and hazards; carefully executed tests of change; and empowerment of team members to work to the full scope of their abilities (Table 1). When information changes frequently, decisions must be able to be revised. Weick describes the risk of decisions becoming like possessions that are justified and defended rather than re-examined.^5^ The purpose of decision-making is to make sense of a situation and provide direction. If the focus is on making sense rather than embracing a decision, it is easier to respond to a dynamic environment. It is possible that personality traits frequent in emergency physicians are well-matched to the demands of a constantly learning HRO. A study of emergency medicine resident personalities compared to resident physician norms found emergency medicine residents to be relatively more “vigilant, team-oriented, flexible, and pragmatic and have a hands-on, practical approach to learning.”^8^

**Table 1.** Principles of high reliability applied to emergency department pandemic response

Principle	Example of emergency department application
Preoccupation with failure	• Regular simulation of critical processes (e.g., protected intubation) • Debriefing after protected resuscitations • Direct observation of personal protective equipment donning and doffing (i.e., buddy system or safety coach) • Clear system of local safety reporting and analysis
Reluctance to simplify	• Awareness of cognitive bias in decision-making • Detailed and nuanced understanding of challenges faced • Thorough analysis of safety events and hazards
Sensitivity to operations	• Gemba walks by leaders • Regularly occurring interprofessional safety huddles • Planned observation of critical processes (e.g., resuscitations) • Use of nimble technologies (e.g., electronic platforms) to rapidly share information
Resilience	• Plan-Do-Study-Act learning in response to challenges • Willingness to modify decisions as information changes • Staff well-being explicitly discussed and addressed
Deference to expertise	• Decision-making by those with most relevant experience as opposed to those with higher rank • Team members using their full scope of knowledge and skills • Leaders recognizing and respecting frontline experience

*Adapted from:* Christianson MK, Sutcliffe KM, Miller MA, et al. Becoming a high reliability organization. Crit Care 2011;15(6):314.

As ED staff prepare to face the demands of operating in pandemic conditions, it is critically important for leaders to understand where high reliability organizing has been effective and where further improvements are required. Here, the quality improvement concept of “Going to the Gemba” is useful. *Gemba* is a Japanese term meaning the “real place.^9^ In Going to the Gemba, leaders visit the place where work is occurring, observe the work, and engage with staff. The strategy, articulated by Toyota Chairman Fujio Cho, is described as “Go see, ask why, show respect.”^10^ Going to the Gemba reduces the likelihood that decision-makers will be guided by incorrect assumptions. Frontline staff are able to describe challenges they may be facing and offer suggestions. Problems are investigated so that effective solutions can be developed. Leaders are able to ensure that staff have the knowledge, resources, and skills they need to carry out processes reliably. Respect is shown to staff by acknowledging their expertise and accepting input in solutions. In the context of pandemic preparation, the act of observing is critical in ensuring that plans are able to be implemented as intended.

We offer as an example of HRO for the COVID-19 pandemic our work in the ED of the Alberta Children's Hospital (ACH). The ACH is a tertiary children's hospital and is part of Alberta Health Services, the healthcare delivery organization that provides acute care for the province of Alberta. While many aspects of pandemic planning occur at the macrosystem and mesosystem level (e.g., capacity-building strategies, procurement of personal protective equipment [PPE]), the details of patient care processes are generally developed at the microsystem level. As an initial step in pandemic planning, our interprofessional ED quality council identified processes in which high reliability is critical. These included resuscitations and intubations, donning and doffing of PPE, and prevention of contamination as staff and patients move through the hospital. Plans for safe and effective processes are developed by an interprofessional team, and individuals with expertise in simulation conduct iterative simulations so that challenges can be recognized and processes can be refined. Strategies are put in place to reduce the likelihood of error in high-risk processes (e.g., standardized workflows, checklists, and direct observation of PPE donning/doffing). ED nursing and physician leaders regularly observe simulations and patient care involving new processes. Trained PPE safety mentors promote sustainability of correct donning/doffing procedures. Staff report challenges, uncertainties, and deviations from best practice, and provide suggestions for improvement, using clear communication channels (e.g., a feedback board in ED, an electronic communication app, and an electronic safety reporting system). Electronic reports of errors, near misses, and hazards are submitted to the safety learning system and reviewed by ED leaders within 24 hours. Questions, challenges, and learnings are summarized and addressed, interprofessional teams assess problems, and Plan-Do-Study-Act cycles are conducted to test changes. Process changes and information are shared daily using electronic platforms. Finally, a safety huddle and brief in situ simulations occur at multiple times of the day to improve situational awareness and ensure understanding of important practices. The intention of these strategies is to use principles of high reliability organizing to improve the safety of staff and patients.

The COVID-19 pandemic will challenge EDs in ways that have never been previously encountered. Staff and patients will face difficulties, challenges, and sometimes heartbreak. Perhaps we may emerge from these struggles stronger and wiser, with a renewed commitment to improving our systems so that we can reduce or eliminate future harm. By incorporating principles of high reliability organizing to our pandemic fight, we can lessen the harm that occurs to ourselves and our patients over the coming months and be more prepared to keep ourselves safe during the future challenges we encounter in our always changing environments.
